# P-1731. Assessing The Impact of an Electronic Health Record Embedded Treatment Algorithm and Order Set on Antipseudomonal Antibiotic Use in Diabetic Foot Infections

**DOI:** 10.1093/ofid/ofae631.1895

**Published:** 2025-01-29

**Authors:** Antoinette M Acbo, Terrence D McSweeney, Hongkai Bao, Kelsie Cowman, Priya Nori, Yi Guo, Mei H Chang

**Affiliations:** Rutgers University / Jersey Shore University Medical Center , Neptune City, NJ; Montefiore Medical Center, Massapequa, New York; Montefiore Medical Center, Massapequa, New York; Montefiore Medical Center, Massapequa, New York; Montefiore Health System, Bronx, NY; Montefiore Medical Center, Massapequa, New York; Montefiore Medical Center, Massapequa, New York

## Abstract

**Background:**

An opportunity to improve diabetic foot infection (DFI) management was identified based on institution-specific epidemiology and antibiotic use (AU) data. *Pseudomonas aeruginosa* prevalence was < 15% upon review of 6,749 unique DFI cultures. However, skin and soft tissue infections were a leading indication for antipseudomonal antibiotics at our institution, driven by DFI. In response, a multidisciplinary team implemented an electronic health record (EHR)-embedded treatment algorithm and order set for DFI treatment.

**Figure:**
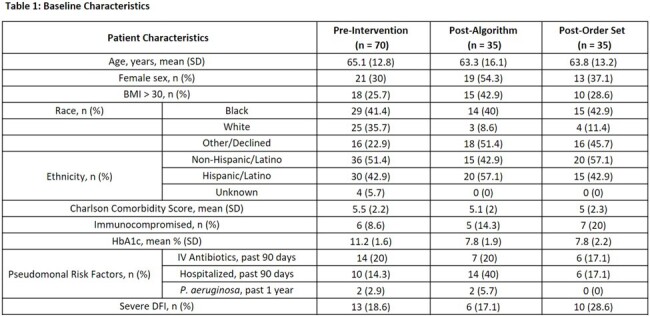

**Methods:**

This multicenter, quasi-experimental study was conducted at an urban, academic healthcare system in the Bronx, NY. Patients were included if they were 18 years of age or older on antibiotics for DFI admitted to vascular surgery or medical floors. Exclusion criteria included duplicate patients, concomitant infection, or transfer from outside hospital. The primary outcome was antipseudomonal AU rate defined as days of therapy (DOT) per 1000 days present. Secondary outcomes included empiric antipseudomonal use, length of stay (LOS), 30-day readmission, 30-day mortality, amputation rates, and *C. difficile* infection (CDI).

**Figure:**
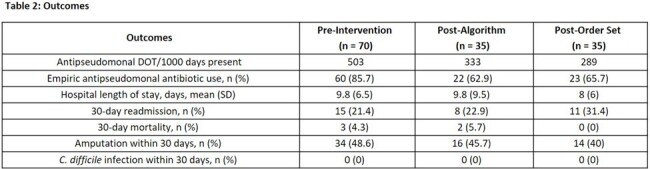

**Results:**

The pre-intervention group included 70 patients, while the post-algorithm and post-order set groups included 35 patients each. Baseline characteristics are listed in Table 1. Antipseudomonal AU was significantly lower in the EHR-embedded treatment algorithm group compared to the pre-intervention group (333 vs 503, p < 0.001); the post-order set group had the lowest antipseudomonal use (289 vs 503, p < 0.001). Empiric antipseudomonal use decreased from 85.7% pre-intervention to 62.9% post-algorithm and 65.7% post-order set (Table 2). Hospital LOS, 30-day mortality, and amputation within 30 days were similar between groups. No CDI occurred. The most frequently isolated bacteria from DFI cultures were *Staphylococcus* aureus and *Streptococcal* species (Figure 1).

**Figure:**
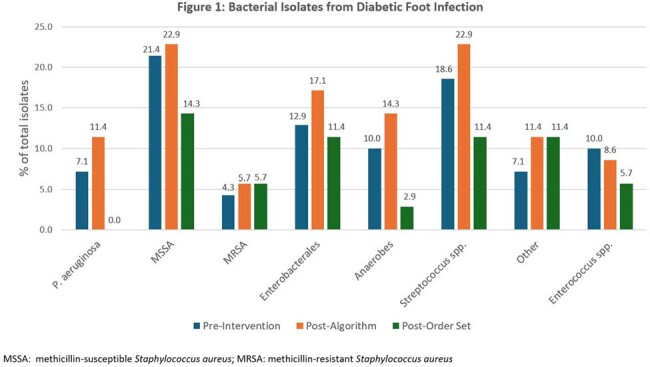

**Conclusion:**

EHR-embedded clinical decision-making tools can effectively reduce antipseudomonal AU for the treatment of DFI without increased risk of 30-day mortality or amputation.

**Disclosures:**

**All Authors**: No reported disclosures

